# A phase I, randomized, single-dose pharmacokinetic study comparing sb8 (bevacizumab biosimilar) with reference bevacizumab in healthy volunteers

**DOI:** 10.1007/s00280-020-04144-7

**Published:** 2020-09-19

**Authors:** Donghoon Shin, Yoon Jung Lee, Jihye Choi, Dahyoung Lee, Minjeong Park, Magdalena Petkova

**Affiliations:** 1 Medical Affairs, Samsung Bioepis Co., Ltd, Incheon, Korea; 2 Clinical Development, Samsung Bioepis Co., Ltd, Incheon, Korea; 3 Biometrics, Samsung Bioepis Co., Ltd, Incheon, Korea; 4 Clinical Bioanalysis, Samsung Bioepis Co., Ltd, Incheon, Korea; 5 Clinical Pharmacology Unit, SGS LSS, Antwerpen, Lange Beeldekensstraat 267, 2060 Antwerpen, Belgium

**Keywords:** SB8, Bevacizumab, Biosimilar, Pharmacokinetics, Immunogenicity

## Abstract

**Purpose:**

To compare pharmacokinetics, safety, tolerability, and immunogenicity between SB8, a bevacizumab biosimilar, and the European Union (EU) and United States (US) reference products (bevacizumab-EU, bevacizumab-US).

**Methods:**

In this randomized, double-blind, parallel-group, and single-dose study, healthy volunteers were randomized to receive a 3 mg/kg dose of SB8, bevacizumab-EU, or bevacizumab-US via intravenous infusion. Primary endpoints were area under the concentration–time curve from time zero to infinity (AUC_inf_) and to the last quantifiable concentration (AUC_last_), and maximum observed serum concentration (*C*_max_). Bioequivalence was achieved if 90% confidence intervals (CIs) for the ratios of the geometric least squares means (LSMeans) of primary endpoints were within the predefined bioequivalence margins of 80.00–125.00%. Safety and immunogenicity were also investigated.

**Results:**

The 90% CIs for the geometric LSMean ratios of AUC_inf_, AUC_last_ and *C*_max_ were all within the prespecified bioequivalence margins. Geometric LSMean ratios for SB8/bevacizumab-EU, SB8/bevacizumab-US and bevacizumab-EU/bevacizumab-US were 88.01%, 88.48% and 100.54% for AUC_inf_, 88.65%, 89.08% and 100.49% for AUC_last_ and 99.59%, 101.15% and 101.56% for *C*_max_, respectively. Incidence of treatment-emergent adverse events (TEAEs) across treatment groups was comparable (SB8: 50.0%, bevacizumab-EU: 37.5%, bevacizumab-US: 53.8%). Most TEAEs were mild and considered as not related to the study drug. No deaths or treatment discontinuations due to adverse events occurred. Incidence of anti-drug antibodies was also comparable between all groups and no neutralizing antibodies were detected.

**Conclusion:**

This study demonstrated pharmacokinetic bioequivalence and similar safety and immunogenicity profiles of SB8 to both reference products, bevacizumab-EU and bevacizumab-US, and of bevacizumab-EU to bevacizumab-US.

**Clinicaltrials.gov identifier:**

NCT02453672 (submitted date); EudraCT number: 2015-001,026-41.

## Introduction

Bevacizumab is a recombinant humanized monoclonal immunoglobulin G1 antibody that inhibits angiogenesis, a hallmark of solid tumor development, by targeting the vascular endothelial growth factor A (VEGF-A) [1]. Bevacizumab was initially approved for treatment of metastatic colorectal cancer by the United States (US) Food and Drug Administration (FDA) (Avastin®, Genentech, Inc., San Francisco, CA, USA) in 2004 and by the European Medicines Agency (EMA) (Avastin®, Roche Pharma AG, Grenzach-Wyhlen, Germany) in 2005. Meanwhile, bevacizumab has been approved in a wide range of oncology indications, including non-small cell lung cancer (NSCLC), metastatic breast cancer (European Union (EU) only), renal cell carcinoma, glioblastoma multiforme (US only), ovarian cancer and cervical cancer [2, 3]. While bevacizumab has demonstrated clinical benefits in terms of prolongation of progression-free and overall survival for patients with advanced cancers [4], access to this treatment option is limited by the high costs of the treatment [5, 6]. Bevacizumab biosimilars may mitigate cost barrier for patients and increase access to an important therapy in oncology.

Biosimilars are biological medicines that are highly similar to an approved biological reference product. Approval of biosimilars by regulatory authorities, such as the US FDA and EMA, follows the so-called “totality of evidence approach”, based on comprehensive demonstration of similarity and therapeutic equivalence to the reference product. This comprises a stepwise approach of extensive comparative analytical and non-clinical assessments, followed by the evaluation of pharmacokinetics (PK), efficacy, safety and immunogenicity in head-to-head, comparative clinical studies of a biosimilar candidate and its reference product(s) [2, 3, 7–9].

The recent expiration of bevacizumab’s patent in the US and EU opened the opportunity for market entry of bevacizumab biosimilars, facilitating competition and enabling the potential for cost-savings for patients and healthcare systems. The first bevacizumab biosimilar, Mvasi (Amgen, Inc., Thousand Oaks, CA, USA), was approved by the US FDA in 2017, followed by the EMA approval in 2018. Subsequently, the second bevacizumab biosimilar, Zirabev (Pfizer Europe MA EEIG, UK), was approved by the US FDA and EMA in 2019 [10–13].

SB8, developed by Samsung Bioepis Co., Ltd., also has been approved as a biosimilar of the reference product bevacizumab by EMA in August 2020 with the brand name of Aybintio, for which analytical and non-clinical similarity have already been established. The primary objective of this phase I study was to demonstrate PK similarity of SB8 to both the EU-sourced (bevacizumab-EU) and US-sourced (bevacizumab-US) reference products, and between bevacizumab-EU and bevacizumab-US, according to EMA and US FDA requirements [2, 3, 7–9, 14]. Secondary objectives were to compare safety, tolerability and immunogenicity between SB8, bevacizumab-EU and bevacizumab-US.

## Materials and methods

### Study population

Eligible participants were healthy males aged 18–55 years with body weight between 65.0 and 90.0 kg and body mass index (BMI) between 20.0 and 29.9 kg/m^2^ at screening and baseline. Participants were excluded if they had a history of and/or current clinically significant gastrointestinal, renal, hepatic, cardiovascular, hematological, pulmonary, neurologic, metabolic, psychiatric, or allergenic (except mild asymptomatic seasonal allergies) diseases. Further key exclusion criteria included a history of arterial thromboembolic events, a history of cancer, unhealed wounds or major surgery planned within 28 days prior to screening or during the study, and alcohol or drug abuse. Participants who have previously been exposed to vascular endothelial growth factor (VEGF) targeting antibody (e.g., bevacizumab, ranibizumab) or any other agent targeting the VEGF receptor (e.g., lapatinib, sunitinib), have been exposed to a biologic agent (other than VEGF-targeting agents) 4 months prior to screening or received another investigational in the context of another clinical study were also excluded.

### Study design

This was a double-blind, single-dose, three-arm, and parallel-group study performed at a single center in Belgium between April 2015 and September 2015 (clinicaltrials.gov identifier: NCT02453672; EudraCT number: 2015-001,026-41). The final study protocol was approved by the Independent Ethics Committee (IEC) of Belgium. This study was conducted in accordance with the ethical principles of the Declaration of Helsinki (1996) that are consistent with International Conference on Harmonisation Good Clinical Practice guidelines (ICH E6) and the applicable local regulatory requirements and laws. The consent documents for the study were reviewed and approved by the IEC prior to use. Investigational products (IPs) were prepared and dispensed by unblinded pharmacists or properly trained pharmacy delegate. IP preparation records were monitored by an unblinded monitor. The subject, the Investigator, site staff, Sponsor, and other study personnel who were involved in the treatment or clinical evaluation of the subjects were unaware of the treatment group assignments.

**Fig. 1:**
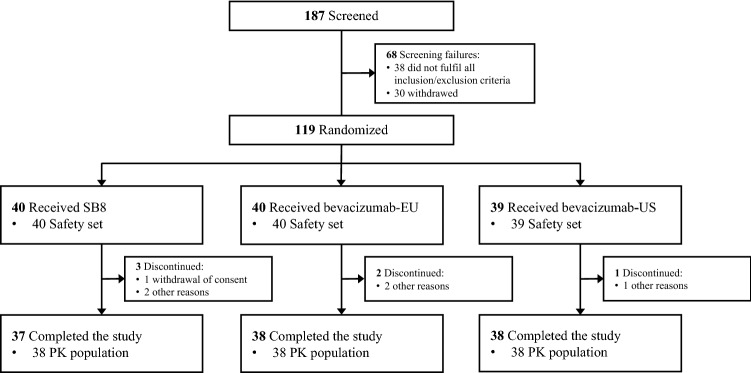
CONSORT diagram of participants flow through the trial. Bevacizumab-EU European Union-sourced bevacizumab, Bevacizumab-US United States-sourced bevacizumab, *PK* pharmacokinetic. Included in PK population since the withdrawal was made between day 71 and day 85 (end of study), after PK blood sampling by day 71

A total of 187 healthy male volunteers were screened within 28 days prior to dosing. Eligible participants were admitted to the clinical research unit on the day before dosing. On day 1, 119 participants were randomized (Fig. 1) to receive a single 3 mg/kg dose of either SB8, bevacizumab-EU, or bevacizumab-US via intravenous (IV) infusion over 90 min, using a single batch for each product. The Investigator and/or nurse were responsible for preparing infusion and setting up the infusion pump. Just before starting the infusion, the Investigator or designated person finally checked whether the dose amount (volume) and infusion time were correctly set on the infusion pump. IP administration by IV infusion for 90 min while subjects were supine or semi-recumbent. Participants were discharged on day 3 and returned to the clinical research unit on an outpatient basis during approximately 12 weeks for PK, safety, and immunogenicity assessments.

### Pharmacokinetic evaluation

Blood samples (approximately 3.5 mL) for PK analysis were collected at 0 (pre-dose), 0.75, 1.5 (end of infusion), 3, 6, 12, 24, 48 and 96 h, then at day 8 (168 h), 15 (336 h), 22 (504 h), 29 (672 h), 43 (1008 h), 57 (1344 h), 71 (1680 h), and 85 (2016 h) after start of infusion. Collected blood samples were clotted for at least 30 min with a clotting activation agent and then centrifuged at 1500–2000 g for approximately 15 min at 4 °C. The serum was stored at  – 60 °C or below until analysis.

PK samples were analyzed at a qualified laboratory using a validated enzyme-linked immunosorbent assay. SB8 or reference bevacizumab in PK samples was captured to the immobilized recombinant human VEGF and unbound material was washed away. Captured SB8 or reference bevacizumab was detected using horseradish peroxidase-labeled anti-human IgG antibody, followed by the addition of tetramethylbenzidine for visualization. Optical density was measured after the color development was stopped and converted to antibody concentration values using StatLIA® (Brendan Bioanalytics, Carlsbad, CA, USA) version 3.2. Serum concentrations below the lower limit of quantification (LLOQ) at pre-dose and in the absorption phase prior to the first quantifiable concentration were considered as zero. PK parameters for analysis of primary and secondary endpoints were calculated based on actual sampling times using a non-compartmental analysis method using Phoenix® WinNonlin® (Certara, St. Louis, MO, USA) version 6.2.

Primary endpoints for PK analysis were AUC_inf_, AUC_last_ and C_max_. The AUC_inf_ was calculated as the area under the concentration–time curve from time zero to the last quantifiable concentration (AUC_last_) plus last observed concentration (*C*_t_)/terminal rate constant (*λ*_z_).

Secondary endpoints for PK analysis were the time at which *C*_max_ was observed (*T*_max_), the volume of distribution during the terminal phase (*V*_z_) calculated as CL/ *λ*_z_ with *λ*_z_ calculated by linear least squares regression analysis using the last 3 (or more) non-zero concentrations, and the terminal half-life (*t*_1/2_) calculated by log_e_(2)/*λ*_z_, the total body clearance (CL) calculated as dose/AUC_inf_.

### Safety evaluation

All adverse events (AEs) including serious adverse events (SAEs) which occurred from screening (days  – 28 to  – 2) until the end of study (EOS, day 85) were recorded. AEs that occurred after study drug administration or that pre-existed and worsened in severity after study drug administration were defined as treatment-emergent adverse events (TEAEs) and were included in the safety analysis.

All AEs were coded according to the Medical Dictionary for Regulatory Activities (MedDRA®) version 18.0, and listed separately by treatment group including participant number, preferred term, seriousness, and severity according to the National Cancer Institute-Common Terminology Criteria for Adverse Events (NCI-CTCAE) version 4.03.

Other safety assessments included clinical laboratory tests (hematology, biochemistry, coagulation, and urinalysis), vital signs, 12-lead electrocardiogram (ECG), and physical examination.

### Immunogenicity evaluation

Blood samples (approximately 10.0 mL) were collected at day 1 (pre-dose), day 22, day 57, and day 85 to determine the incidence and titers of anti-drug antibodies (ADAs) and the incidence of neutralizing antibodies (NAbs). A post-dose ADA result was defined as positive if at least one of the post-baseline (day 22, day 57 or day 85) ADA tests was positive.

Immunogenicity samples were analyzed at a qualified laboratory using validated methods with a multi-tiered approach, consisting of a screening and a confirmatory assay for ADAs, as well as a titer and a neutralization assay. Binding ADAs were detected using a qualitative and semiquantitative electrochemiluminescent assay. In ADA-positive response samples, the NAbs were detected using an electrochemiluminescent assay.

### Statistical methods

Sample size was determined based on known variability of PK parameters in cancer patients. A pooled interparticipant percent coefficients of variation (%CV) of 31% was derived based on the %CV for AUC_inf_ in patients with solid tumors, which are 26%, 55%, and 19% after 1 mg/kg, 3 mg/kg, and 10 mg/kg bevacizumab administration, respectively [15]. As less variability in PK parameters is expected in healthy volunteers, a %CV of 25% was used for sample size calculation based on the assumption that the %CV in healthy participants is approximately 80% of that in patients with solid tumors. The required sample size was calculated to achieve sufficient statistical power to reject the null hypotheses that the lower and upper limits of the 90% CI for the ratio of the geometric means between the test and reference were below 80.00% and above 125.00%, respectively. Assuming an expected ratio of means of 1.05 for AUC_inf_, an estimated 36 participants per treatment group are required to achieve 90% power using two one-sided t-tests, each at the 5% significance level. With an assumed 5% dropout rate, this resulted in an estimate of 38 participants to be randomized in each treatment group.

The safety set included all participants who were randomized and received the study drugs and was used for the analysis of safety and immunogenicity. PK analysis was performed in the pharmacokinetics population, which included all participants in the safety set without any major protocol deviation.

Bioequivalence for AUC_inf_, AUC_last_, and *C*_max_ was determined if the 90% CI for the ratio of geometric least squares means (LSMeans) of SB8 to bevacizumab-EU, SB8 to bevacizumab-US, and bevacizumab-EU to bevacizumab-US was within the predefined bioequivalence margins of 80.00–125.00%. For the calculation of PK parameters, no imputations were carried out in case of missing date and/or time, considering a worst-case consideration (e.g. record allocation to a ‘worst’ analysis period).

The statistical analysis of the log_e_-transformed primary endpoint was performed by the analysis of variance (ANOVA) model with treatment as a fixed effect. The difference in geometric LSMeans between SB8 and bevacizumab-EU, SB8 and bevacizumab-US, as well as bevacizumab-EU and bevacizumab-US and the corresponding 90% CIs were determined. Back transformation provided the ratio of geometric LSMeans and 90% CI for these ratios.

## Results

### Participants

A total of 119 male participants received a single dose of study drug (SB8, *n* = 40; bevacizumab-EU, *n* = 40; bevacizumab-US, *n* = 39), of which 113 completed the study (Fig. 1). Six participants discontinued due to withdrawal of informed consent (*n* = 1, 0.8%) and other reasons (*n* = 5, 4.2%). None of the participants discontinued the study due to an AE. Five participants (SB8, *n* = 2; bevacizumab-EU, *n* = 2; bevacizumab-US, *n* = 1) were excluded from the primary PK analysis due to major protocol deviation (Fig. 1). Major protocol deviations were reported for subjects meeting exclusion criteria (2 subjects), not meeting inclusion criteria (1 subject), non-compliance with the IP dosage (1 subject), and taking disallowed therapy during the treatment period (1 subject). The final per-protocol population used in the PK analysis consisted of 114 participants (SB8, *n* = 38; bevacizumab-EU, *n* = 38; bevacizumab-US, *n* = 38) (Fig. 1). Demographics and other baseline characteristics were comparable between the treatment groups (Table 1).

**Table 1:** Demographics and other baseline characteristics (Randomized set)

Parameter	SB8 (*N* = 40)	Bevacizumab-EU (*N* = 40)	Bevacizumab-US (*N* = 39)	Total (*N* = 119)
Ethnicity, *n* (%)^a^				
Caucasian	37 (92.5)	37 (92.5)	38 (97.4)	112 (94.1)
Black or African American	1 (2.5)	2 (5.0)	1 (2.6)	4 (3.4)
Asian	1 (2.5)	1 (2.5)	0 (0.0)	2 (1.7)
American Indian or Alaska Native	1 (2.5)	0 (0.0)	0 (0.0)	1 (0.8)
Age (years)	44.2 ± 9.24	39.5 ± 10.45	38.9 ± 9.32	40.9 ± 9.90
Height (cm)	178.42 ± 5.682	176.49 ± 5.159	177.79 ± 6.832	177.56 ± 5.929
Weight (kg)	79.90 ± 6.639	78.97 ± 6.647	80.02 ± 6.259	79.63 ± 6.481
BMI (kg/m^2^)	25.14 ± 2.347	25.39 ± 2.249	25.38 ± 2.294	25.30 ± 2.281

Data are presented in mean ± SD unless otherwise indicated

Bevacizumab-EU bevacizumab sourced from the European Union, Bevacizumab-US bevacizumab sourced from the United States, *BMI* body mass index, *N* number of randomized participants, n number of participants with available result, *SD* standard deviation

^a^Percentages are based on the number of randomized participants

### Pharmacokinetics

**Fig. 2:**
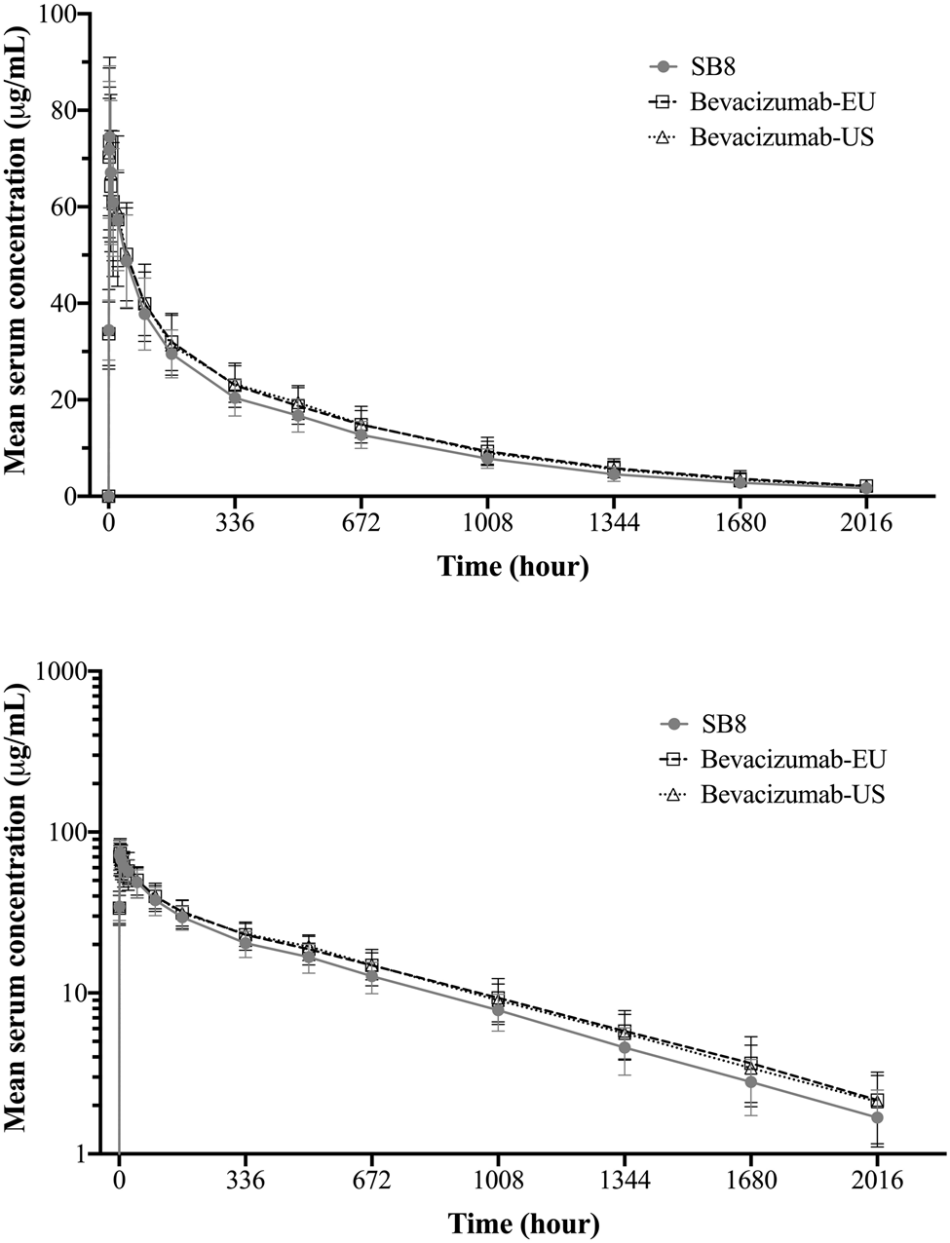
Mean bevacizumab serum concentrations versus nominal times on linear and semi-logarithmic scale (PK population). Mean serum concentrations versus nominal times on linear (top graph) and semi-logarithmic scale (bottom graph) of SB8, bevacizumab-EU, and bevacizumab-US. Graphs were made with GraphPad Prism (GraphPad Software, La Jolla, CA, USA) version 8.4.1. Bevacizumab-EU: bevacizumab sourced from the European Union, Bevacizumab-US: bevacizumab sourced from the United States

The mean serum concentration–time profiles were superimposable between SB8, bevacizumab-EU and bevacizumab-US (Fig. 2). Maximum serum concentrations were reached between 1.5 and 3 h after the start of infusion and were followed by a rapid decrease in serum concentrations and a subsequent slow elimination phase. Both maximum and overall exposure were similar across the treatment groups with t_1/2_ ranging between 18.5 and 19.3 days (Table 2). The 90% CIs of the geometric LSMeans ratios for AUC_inf_, AUC_last_, and *C*_max_ were fully contained within the pre-defined bioequivalence margin of 80.00–125.00% for all comparisons between treatment groups (Table 3).

**Table 2:** Summary of PK parameters (PK population)

Parameter	SB8 (*N* = 38)	Bevacizumab-EU (*N* = 38)	Bevacizumab-US (*N* = 38)
AUC_inf_ (h·μg/mL)	25,354.4 ± 4833.10	28,896.8 ± 6221.62	28,684.8 ± 5425.14
AUC_last_ (h·μg/mL)	24,199.2 ± 4367.53	27,342.2 ± 5374.53	27,177.9 ± 4770.93
*C*_max_ (μg/mL)	76.259 ± 14.6999	76.059 ± 11.7053	76.485 ± 16.9916
*T*_max_ (hour)	3.000 (1.52, 12.00)	3.000 (1.52, 12.00)	3.000 (1.52, 97.12)
*t*_1/2_ (hour)	444.4 ± 79.46	464.2 ± 81.06	462.8 ± 86.98
*V*_z_ (mL)	6,118.6 ± 960.98	5,566.4 ± 833.62	5,654.1 ± 999.97
CL (mL/hour)	9.721 ± 1.7803	8.517 ± 1.6570	8.659 ± 1.8600

Data are presented in mean ± SD except *T*_*max*_ which is presented in median (min, max)

*AUC*_*inf*_ area under the concentration–time curve from time zero to infinity, *AUC*_*last*_ area under the concentration–time curve from time zero to the last quantifiable concentration, Bevacizumab-EU bevacizumab sourced from the European Union, Bevacizumab-US bevacizumab sourced from the United States, CL total body clearance, *C*_*max*_ maximum observed serum concentration, N number of participants in the PK population, SD standard deviation, *T*_*max*_ time at which *C*_*max*_ was observed, *t*_*1/2*_ terminal half-life, *V*_*z*_ volume of distribution during the terminal phase

**Table 3:** Statistical comparison of primary PK parameters between test and reference products (PK population)

Test	Reference	PK parameter	Geometric LS mean		Ratio (%) (90% CI)
			Test	Reference	
SB8 (*N* = 38)	Bevacizumab-EU (*N *= 38)	AUC_inf_ (h·μg/mL)	24,901.3	28,294.9	88.01 (81.54, 94.98)
		AUC_last_ (h·μg/mL)	23,812.9	26,862.9	88.65 (82.58, 95.16)
		*C*_max_ (μg/mL)	74.927	75.232	99.60 (93.33, 106.28)
SB8 (*N* = 38)	Bevacizumab-US (*N* = 38)	AUC_inf_ (h·μg/mL)	24,901.3	28,143.3	88.48 (82.01, 95.46)
		AUC_last_ (h·μg/mL)	23,812.9	26,732.5	89.08 (82.96, 95.65)
		*C*_max_ (μg/mL)	74.927	74.074	101.15 (92.23, 110.93)
Bevacizumab-EU (*N* = 38)	Bevacizumab-US (*N* = 38)	AUC_inf_ (h·μg/mL)	28,294.9	28,143.3	100.54 (92.99, 108.70)
		AUC_last_ (h·μg/mL)	26,862.9	26,732.5	100.49 (93.49, 108.01)
		*C*_max_ (μg/mL)	75.232	74.074	101.56 (93.13, 110.76)

*AUC*_*inf*_ area under the concentration–time curve from time zero to infinity, *AUC*_*last*_ area under the concentration–time curve from time zero to the last quantifiable concentration, Bevacizumab-EU bevacizumab sourced from the European Union, Bevacizumab-US bevacizumab sourced from the United States, *CI* confidence interval, *C*_*max*_ maximum serum concentration, *LS* least square, *PK* pharmacokinetic, *N* number of participants in the PK population

### Safety

A total of 85 TEAEs were reported in 56 (47.1%) participants. Overall, 32 TEAEs were reported in 20 (50.0%) participants in the SB8 group, 17 TEAEs were reported in 15 (37.5%) participants in the bevacizumab-EU group, and 36 TEAEs were reported in 21 (53.8%) participants in the bevacizumab-US group (Table 4). Overall, the majority of TEAEs were mild (SB8 *n* = 19, 47.5%; bevacizumab-EU *n* = 15, 37.5%; bevacizumab-US *n* = 20, 51.3%) in severity and were not related to the study drug. The most common TEAEs were diarrhea (*n* = 4, 10.0%) in the SB8 group, nasopharyngitis (*n* = 6, 15.0%) in the bevacizumab-EU group, and headache (*n* = 7, 17.9%) and nasopharyngitis (*n* = 6, 15.4%) in the bevacizumab-US group; all other TEAEs were reported in < 10.0% of subjects per treatment group. There were no deaths or discontinuations due to TEAEs during the study. One SAE, a case of perirectal abscess, occurred on day 53 post-dose in the SB8 group (2.5%). This SAE resolved by the end of the study following treatment with trans-anal drainage and antibiotics and was considered as not related to the study drug. Clinical laboratory data, vital signs, and 12-lead ECG parameters did not show any clinically relevant changes over time.

**Table 4:** Summary of TEAEs (Safety set)

Category	SB8 (*N* = 40) *n* (%)	Bevacizumab-EU (*N* = 40) *n* (%)	Bevacizumab-US (*N* = 39) *n* (%)	Total (*N* = 119) *n* (%)
Any AE	23 (57.5)	15 (37.5)	22 (56.4)	60 (50.4)
Any TEAE	20 (50.0)	15 (37.5)	21 (53.8)	56 (47.1)
Any SAE	1 (2.5)^a^	0 (0.0)	0 (0.0)	1 (0.8)^a^
*TEAE severity*				
Grade 1	19 (47.5)	15 (37.5)	20 (51.3)	54 (45.4)
Grade 2	0 (0.0)	0 (0.0)	0 (0.0)	0 (0.0)
Grade 3	1 (2.5)	0 (0.0)	1 (2.6)	2 (1.7)
Grade 4 or 5	0 (0.0)	0 (0.0)	0 (0.0)	0 (0.0)
*TEAE causality*				
Not related	19 (47.5)	14 (35.0)	18 (46.2)	51 (42.9)
Related	1 (2.5)	1 (2.5)	3 (7.7)	5 (4.2)
*TEAEs occurring in ≥ 5% of participants by PT*				
Nasopharyngitis	3 (7.5)	6 (15.0)	6 (15.4)	15 (12.6)
Headache	3 (7.5)	0 (0.0)	7 (17.9)	10 (8.4)
Diarrhea	4 (10.0)	2 (5.0)	0 (0.0)	6 (5.0)
Back pain	2 (5.0)	1 (2.5)	2 (5.1)	5 (4.2)
Oropharyngeal pain	2 (5.0)	0 (0.0)	2 (5.1)	4 (3.4)

*AE* adverse event, Bevacizumab-EU bevacizumab sourced from the European Union, Bevacizumab-US bevacizumab sourced from the United States, *IP* investigational product, *PT* preferred term, *N* number of participants in the Safety set, *n* number of subjects with that observation, *SAE* serious AE, *TEAE* treatment-emergent AE

Percentages are based on the number of participants in the safety set. If a participant had multiple events with different severity (or causality), then the participant was counted only once at the worst severity (or causality)

^a^Perirectal abscess in participant 01,204 (SB8 group)

### Immunogenicity

The overall incidence of participants with post-dose ADA-positive result was 1/39 (2.6%) in the SB8 group, 4/39 (10.3%) in the bevacizumab-EU group and 1/38 (2.6%) in the bevacizumab-US group, respectively. There was no statistically significant difference in post-dose ADA incidence across the 3 treatment groups (SB8 vs bevacizumab-EU, *p* = 0.3584; SB8 vs bevacizumab-US, *p* = 1.0000; bevacizumab-EU vs bevacizumab-US, *p* = 0.3584). Five participants in whom ADAs were detected on day 22 were subsequently ADA-negative on day 57 and by the end of study, illustrating their transient nature of these ADAs. In the one remaining ADA-positive case, ADA was only detected in the SB8 group by the end of study. None of the participants had a positive result for NAbs.

## Discussion

This is the first clinical study of SB8, an approved bevacizumab biosimilar. The results of this study demonstrate bioequivalence of SB8 to both reference products, EU-sourced and US-sourced bevacizumab, for all tested PK parameters. Moreover, results confirm that bevacizumab-EU and bevacizumab-US are bioequivalent to each other, consistent with results from other phase I studies of bevacizumab biosimilars [16, 17]. The PK results of this study are consistent with those of other phase I PK studies of (proposed) bevacizumab biosimilars which also demonstrated equivalence to the reference product [16–26]. However, direct comparisons of absolute PK parameters values between studies are generally not appropriate due to differences between the doses used (1 mg/kg, 3 mg/kg or 5 mg/kg), sample collection, and assessment methods [16–26].

Due to the long half-life of bevacizumab (approximately 20 days) and the potential influence of immunogenicity on PK parameters, a parallel-group rather than a cross-over design was considered appropriate. To ensure the assessment of potential differences in PK profiles in a sensitive population, which is essential for the demonstration of bioequivalence [2, 3, 14], this study was performed in healthy male volunteers. In patients with cancer, potential confounding factors, such as varying disease stage and prognosis, tumor burden, disease-specific complications, comorbidities, and concomitant medications, may increase variability in PK parameters and mask potential differences between treatment groups. Females have been excluded because a potential risk of fertility impairment due to ovarian failure associated with exposure to bevacizumab was reported [27, 28]. In addition, variability due to gender difference in PK profiles of bevacizumab, as male cancer patients have 20% increase in central volume and 17% higher clearance compared to female patients, was considered [15]. The approved therapeutic doses of bevacizumab for treatment of cancer range from 5 to 10 mg/kg every 2 weeks to 15 mg/kg every 3 weeks. Given the known linearity of bevacizumab PK in the 1 to 10 mg/kg dose range [27, 28], a single dose of 3 mg/kg was chosen to minimize exposure in healthy volunteers, while still obtaining meaningful PK results considering the LLOQ of the applied method for drug concentration measurement. Assessment of pharmacodynamics was not considered in this study since clinically applicable predictive biomarker of bevacizumab has not been identified [29].

Safety profiles were similar between the treatment groups, with no observed clinically meaningful differences. SB8 was well-tolerated and no safety concerns were identified. Consistently, no safety concerns were identified from Phase III study with SB8 in in patients with NSCLC, with administration of 15 mg/kg SB8 every 3 weeks for 24 weeks in combination with chemotherapy, followed by SB8 maintenance monotherapy [30]. Immunogenicity was comparable between all treatment groups and no NAbs were detected.

In summary, this phase I study demonstrated that SB8 is bioequivalent to the reference product bevacizumab, with similar safety and immunogenicity profiles in healthy volunteers. These results represent an important contribution to the totality of evidence for supporting the biosimilarity of SB8 to its reference product bevacizumab, following the demonstration of analytical and functional similarity in extensive quality and non-clinical assessments. This totality of evidence is complemented by further clinical data from a phase III study in patients with NSCLC, demonstrating equivalence of SB8 and bevacizumab in terms of best overall response rate and comparable safety, PK and immunogenicity [30].

## Data Availability

Upon request, and subject to certain criteria, conditions, and exceptions, Samsung Bioepis will provide access to individual de-identified participant data to researchers whose proposals meet the research criteria and other conditions and for which an exception does not apply. Proposals should be directed to the corresponding author. For access, data requestors must enter into a data access agreement with Samsung Bioepis.
